# Routine serum creatinine measurements: how well do we perform?

**DOI:** 10.1186/s12882-015-0012-x

**Published:** 2015-02-14

**Authors:** Liesbeth Hoste, Kathleen Deiteren, Hans Pottel, Nico Callewaert, Frank Martens

**Affiliations:** Interdisciplinary Research Facility Life Sciences, Katholieke Universiteit Leuven Campus Kortrijk, Kortrijk, Belgium; Department of Clinical Chemistry and Toxicology, AZ Groeninge Hospital, Reepkaai 4, B8500 Kortrijk, Belgium

**Keywords:** Creatinine, External quality assessment, Glomerular filtration rate

## Abstract

**Background:**

The first aim of the study was to investigate the accuracy and intra-laboratory variation of serum creatinine measurements in clinical laboratories in Flanders. The second purpose was to check the effect of this variation in serum creatinine concentration results on the calculated estimated glomerular filtration rate (eGFR) and the impact on classification of patients into a chronic kidney disease (CKD) stage.

**Methods:**

26 routine instruments were included, representing 13 different types of analyzers from 6 manufacturers and covering all current methodologies (Jaffe, compensated Jaffe, enzymatic liquid and dry chemistry methods). Target values of five serum pools (creatinine concentrations ranging from 35 to 934 μmol/L) were assigned by the gold standard method (ID-GC/MS).

**Results:**

Intra-run CV (%) (n = 5) and bias (%) from the target values were higher for low creatinine concentrations. Especially Jaffe and enzymatic dry chemistry methods showed a higher error. The calculated eGFR values corresponding with the reported creatinine concentration ranges resulted in a different CKD classification in 47% of cases.

**Conclusions:**

Although most creatinine assays claim to be traceable to the gold standard (ID-GC/MS), large inter-assay differences still exist. The inaccuracy in the lower concentration range is of particular concern and may lead to clinical misinterpretation when the creatinine-based eGFR of the patient is used for CKD staging. Further research to improve harmonization between methods is required.

**Electronic supplementary material:**

The online version of this article (doi:10.1186/s12882-015-0012-x) contains supplementary material, which is available to authorized users.

## Background

The calculation of the estimated glomerular filtration rate (eGFR) using mathematical formulas has been encouraged as a simple, rapid and reliable way of assessing kidney function. A problem related to the use of creatinine-based eGFR formulas is the existing diversity of methods to determine serum creatinine (Scr). Small analytic changes in Scr can create major shifts in the distributions of eGFR, which then cause large differences in the eGFR-based chronic kidney disease (CKD) classification of patients [1]. So it is clear that control of laboratory analysis of Scr and worldwide standardized Scr measurements are necessary. According to the National Kidney Disease Education Program (NKDEP) recommendations, manufactures already made efforts to standardize their Scr measurements to have calibration traceable to the isotope dilution gas chromatography/mass spectrometry (ID-GC/MS) gold standard method. However calibration traceability does not address non-specificity which remains of concern.

In the Jaffe reaction creatinine forms a coloured product after addition of alkaline picrate. However, proteins, glucose and substances with a ketone group are known to interfere [2]. Many manufacturers tried to improve the performance characteristics of the Jaffe reaction by compensating for these interferences (eg. rate blanking and subtraction of a fixed factor to compensate for non-specific reactions). Since 1970 enzymatic assays were developed to improve creatinine specificity. These enzymatic methods have generally fewer interferences than the Jaffe methods and therefore result in more accurate staging of CKD [3]. The higher cost of the enzymatic Scr assay is the main reason why the use of Jaffe or compensated Jaffe assays is still in practice [4]. Recently Piéroni et al. demonstrated substantial improvement in the calibration, traceability and precision of the enzymatic methods, reaching the NKDEP recommendations [5].

The importance of standardized creatinine measurements to calculate eGFR in a correct way has also led to the development of improved creatinine-based eGFR equations. Two of the most popular eGFR formulas, the original pediatric Schwartz equation [6] and the original MDRD (Modification of Diet in Renal Disease) [7] equation, were derived using Scr levels measured by the kinetic Jaffe method. This method is known to overestimate ID-GC/MS-traceable Scr up to 20%. After the widespread standardization of creatinine measurement methods both equations were re-expressed for ID-GC/MS standardized (compensated Jaffe or enzymatic) Scr [8-10]. Also the popular CKD-EPI (Chronic Kidney Disease Epidemiology Collaboration) equation [11] was developed to be used only with ID-GC/MS standardized Scr. Unfortunately, the restricted use of these formulas for specific creatinine methods has not been an overriding concern in some studies.

Our study was performed after the widespread standardization of creatinine measurement methods and reports on the status of standardization in clinical laboratories in Belgium – Flanders. The first aim of the present study was to investigate the intra-run variation and accuracy (bias) of commonly used Scr assays in Flanders. It was our goal to include all current methodologies including Jaffe, compensated Jaffe and enzymatic assays (dry or liquid chemistry). Our interest was to study how results can vary if patients are followed in different laboratories that work with various assays or instruments eg. in the lab of the general practitioner or in the lab of the specialized doctor in the hospital. The second purpose was to investigate the effect of the variation in Scr determination on eGFR values and on the CKD classification for some specific patient cases. Five fresh frozen serum pools were prepared in a large concentration range (35 to 934 μmol/L) by minimal processing. Therefore the properties of the serum pools are comparable with authentic clinical samples (commutable). This is a major advantage over the non-commutable control materials (eg. lyophilized) typically used in (inter)national proficiency schemes. In contrast to the study of Piéroni et al. [5], we specified that the routine settings were used instead of performing calibration just before the run of the study samples. The participants were also asked to report the results like they usually do. This approach mirrors the best the actual situation.

## Methods

### Preparation of serum pools

The creatinine samples of different concentrations were prepared in AZ Groeninge Hospital Kortrijk, Belgium by pooling fresh leftovers of patient serum samples. The samples of coagulated blood were centrifuged (10 min at 3000 rpm) immediately after arrival in the laboratory. The creatinine concentration was determined enzymatically on a Cobas 6000 (Roche) and the samples were stored at 4°C (max 3 days). According to the creatinine concentration the serum was added to a specific frozen pool (n = 5) and allowed to freeze at −20°C on top of the already frozen material. All pools were prepared within one week. Once the needed volume was reached, the pools were thawed at room temperature and homogenized (30 min, roller mixer). Afterwards the thawed pools were centrifuged (10 min, 3000 rpm). No pellet was visible.

The combined serum samples were tested for viral serology and found negative for hepatitis B surface antigen, hepatitis C virus, human immunodeficiency virus and syphilis. In all pools bilirubin was <53 μmol/L, triglycerides <1.92 mmol/L and no hemolysis was observed [12]. One mL aliquots of the homogenized pools were frozen at −80°C until shipping on dry ice to the reference laboratory (ID-GC/MS) and to the 22 participating clinical laboratories.

The study was approved by the local Ethical Committee of the AZ Groeninge Hospital Kortrijk, Belgium (reference number B39620140695). Since the pools were prepared using remains of patient serum samples that were anonymized, it was not necessary to obtain written informed consent.

### Target assignment of serum pools

Because the ID-GC/MS method is believed to be without interference of other substances it is considered the best method for determining creatinine concentrations. The target values were assigned by an ID-GC/MS reference method approved by the international Joint Committee on Traceability in Laboratory Medicine (JCTLM) (Department of Analytical Chemistry of Prof. Dr. Thienpont at the University of Ghent, Belgium) [13,14]. Since it is a highly laborious and costly method, only a few highly specialized laboratories worldwide are offering this method. The measurement protocol consisted of analysis of each sample in triplicate on 3 independent occasions (separate sampling, sample preparation, independent calibration and mass-spectrometry measurement). Calibration mixtures were always prepared from three independently prepared working solutions. The expanded uncertainty (k = 2) for that measurement protocol was estimated to be 2.8%.

### Assessment of serum pools

We set up an experiment to test whether the freeze-thaw cycle used to prepare the serum pools had altered the properties of creatinine and thus commutability. Over a period of three days, the same volume of serum obtained from patient samples (n = 23) was added to a liquid pool that was saved in the fridge at 4°C and separately to a pool that was kept frozen at −20°C. Afterwards, 30 aliquots were taken from the liquid pool and from the defrosted pool respectively and analyzed for Scr in one run on the Cobas 6000 (Roche). The sample size of n = 30 for this experiment was obtained from Pass^12^, NCSS statistical software (Kaysville, Utah, USA), based on the following: an equivalence test of means using two one-sided tests on data from a parallel-group design with sample sizes of 30 in the liquid pool and 30 in the defrosted pool achieves 90% power at a 5% significance level when the true difference between the means is 1.24 μmol/L (the maximum allowed difference that is considered equivalent), the standard deviation is 1.00 μmol/L and the equivalence limits are −2.00 and 2.00 μmol/L.

### Serum creatinine measurements

The pools were shipped on dry ice to the 22 participating labs (14 hospital laboratories and 8 non-hospital laboratories) and were measured within one month after arrival. In the meantime, the pools were kept frozen in the participant’s laboratory at −20°C. The participants analyzed each pool in 5-fold on their automated chemistry analyzer in routine and within 3 hours after thawing of the samples. The participants delivered information on the instrument, reagents and the method applied. The study included 26 routine methods, representing 13 different types of analyzers from 6 manufacturers covering all current methodologies. Serum creatinine was measured either enzymatically (E), liquid or dry or by a Jaffe method, alkaline picrate kinetic (AP-K) or with an alkaline picrate rate-blanked method (AP-RB) with compensation (C) or without compensation (NC) (Table 1). In a dry chemistry assay, the sample is deposited onto a multi-layered slide and evenly distributes into the slide that contains all appropriate substrates and other components for a reaction. A liquid assay takes place in a liquid environment. The AP-RB method was developed to reduce bilirubin interference. The rate of colour change in the sample at alkaline pH is used as a correction factor for the rate observed after the addition of picric acid.

**Table 1:** **Characteristics of methods used by the participants**

	**Instrument**			
**Manufacturer**	**Analyzer**	**Reagent**	**No.**	**Method**
Beckman Coulter Inc, Brea, CA, USA	LX 20 Clinical System (Synchron)	CREm (Creatinine) Reagent	n = 1	AP-K, NC
	UniCel DxC 800 Synchron Clinical System	CREm (Creatinine) Reagent	n = 1	AP-K, NC
Roche Diagnostics, Mannheim, Germany	Modular P	CREA Creatinine Jaffe method compensated	n = 2	AP-RB, C
	Integra 400 plus	Creatinine Jaffé Gen.2 compensated, CREP2 Creatinine plus vers.2	n = 2	AP-K, C and E liquid
	Integra 800	Creatinine Jaffé Gen.2 compensated, CREP2 Creatinine plus vers.2	n = 3	AP-K, C and E liquid
	Cobas 6000	Creatinine Jaffé Gen.2 compensated, CREP2 Creatinine plus vers.2	n = 6	AP-K, C and E liquid
	Cobas 8000	Creatinine Jaffé Gen.2 compensated	n = 2	AP-RB, C
Abbott Laboratories, Abbott Park, Il, USA	Architect C16000	CreaC	n = 2	AP-K, NC
Siemens, Erlangen, Germany	Dimension Vista 1500	ECREA, CREA: Dimension Vista creatinine	n = 2	E liquid or AP-K, NC
	Advia 1650	CREA	n = 1	AP-K, NC
Sysmex Europe, Norderstedt, Germany	Olympus AU2700	Creatinine Jaffe	n = 1	AP-K, NC
Ortho Clinical Diagnostics (OCD) Inc, Rochester, NY, USA	Vitros 5600	CREA slides	n = 1	E dry
	Vitros 5,1 FS	CREA slides	n = 2	E dry

AP-K = alkaline picrate kinetic; AP-RB = alkaline picrate rate-blanked; C = compensated; NC = non-compensated; E = enzymatic.

One participant included four of their routine Cobas 6000 (Roche) instruments in the study, another participant included two Integra 800 (Roche) instruments. The laboratory working with the LX 20 Clinical System (Jaffe) and the laboratory working with the Dimension Vista 1500 (Jaffe) reported their results (in mg/dL) accurate to the nearest tenth, while all the other laboratories reported results accurate to the nearest hundredth.

### eGFR calculations

To estimate the effect of the variation in Scr determinations on the eGFR, we calculated eGFR values corresponding with the minimum and maximum determined Scr values of each pool for some specific patient cases. The first pool (35 μmol/L) corresponded with the average Scr value of a six year old healthy child with a length of 116.5 cm. There are multiple eGFR equations to calculate the GFR in children [6,8,15,16]. We used the Schwartz equation which is nowadays still the most used formula to calculate the eGFR in the pediatric population [6,8]. Although the minimum and maximum Scr values for this pool were determined with Jaffe type assays we used the updated Schwartz formula [eGFR = 0.413 * length (cm)/ Scr (mg/dL)] [8], since the original Schwartz formula [6] has been validated 30 years ago using Jaffe recipes that are no longer on the market [17]. For pool 2 to 5, we used the CKD-EPI equation [eGFR = 141 * min(Scr/κ,1)^α^ * max(Scr/κ,1)^-1.209^ * (0.993)^Age^ * (1.018 if female) * (1.159 if black) where Scr expressed in mg/dL and κ = 0.90 mg/dL for males and 0.70 mg/dL for females; α = −0.411 for males and −0.329 for females] to calculate the eGFR for both an 18 year as well as a 65 year old woman and man [11]. To convert Scr from the SI-unit (μmol/L) to the conventional unit (mg/dL), the SI-unit must be divided by the conversion factor 88.4. The corresponding CKD stages (Grade (G) 1–5) can be found in Table 2.

**Table 2:** **eGFR calculations and subsequent CKD classification based on minimum and maximum serum creatinine results obtained for each pool**

**Pool, μmol/L**		**1**		**2**		**3**		**4**		**5**	
		**35**		**70**		**112**		**296**		**934**	
**Min and max Scr, μmol/L**		**27**	**53**	**59**	**80**	**104**	**124**	**274**	**313**	**868**	**990**
**eGFR, ml/min/1.73 m** ^**2**^	**child 6y**	158	80^a,b^								
	CKD stage	G1	G2								
	**man 18y**			140	124	90^a,b^	73	28	24	7	6
	CKD stage			G1	G1	G1	G2	G4	G4	G5	G5
	**woman 18y**			128	93	68	55^a,b^	21	18	5	4
	CKD stage			G1	G1	G2	G3a	G4	G4	G5	G5
	**man 65 y**			101	89^a,b^	65^a,b^	52	20	17	5	4
	CKD stage			G1	G2	G2	G3a	G4	G4	G5	G5
	**woman 65y**			92^a,b^	67	49^a,b^	39	15	13^a,b^	4	3
	CKD stage			G1	G2	G3a	G3b	G4	G5	G5	G5

The updated Schwartz formula was used for eGFR calculation in the 6 year old child with a length = 116.5 cm [8]. The CKP-EPI formula was used for eGFR calculations in adults [11] (see Methods section).

(Grade 1) Normal GFR: ≥90 mL/min/1.73 m^2^, (Grade 2) Mild impairment: 60-89 mL/min/1.73 m^2^, (Grade 3a) Mild to moderate impairment: 45-59 mL/min/1.73 m^2^, (Grade 3b) Moderate to severe impairment: 30-44 mL/min/1.73 m^2^, (Grade 4) Severe impairment: 15-29 mL/min/1.73 m^2^ and (Grade 5) End stage renal disease: <15 mL/min/1.73 m^2^. In the absence of kidney damage, Grade 1 and 2 cannot be considered as CKD, but Grade 3 is always considered as CKD.

^a^Indicates cases for which eGFR calculated for the minimum and maximum serum creatinine values classify patients in different CKD stages.

^b^Indicates cases for which the calculated CKD stage is different from the CKD stage obtained when the target value of the pool is used.

### Statistical analysis

For each instrument, the mean of the 5 measurements of each pool and the standard deviation (SD) were calculated. Analytical imprecision (i.e. the degree of agreement of replicate measurements) of each analyzer was assessed by calculating the intra-run coefficient of variation (CV_i_)(%) (n = 5). Also the bias (B)(%) was calculated as [(measured Scr – ID-GC/MS target value Scr)/ ID-GC/MS target value Scr]*100. CV_i_ and bias were compared to the performance specifications for creatinine as reported in the Ricos-Fraser et al. database (Additional file 1) [18]. Finally, an error (1.65CV_i_ + B) was calculated and compared to the Ricos-Fraser total error (TE) to estimate the leftover budget for the not known inter-run CV (inter-run experiments not performed). The Ricos-Fraser database is based on the within- and between subject variation of laboratory parameters. The database defines the insufficient, minimal, desirable and optimal analytical precision, bias and total error of an analytical assay (eg. for creatinine see Additional file 1).

## Results

### Experimental design

The concentrations of the five creatinine serum pools were chosen carefully. Pool 1 was designed to correspond to the average Scr concentration of a six year old child (around 35 μmol/L) [15]. Pool 2 was designed to be in the pathological Scr range for children or within the normal adult creatinine concentration range (around 70 μmol/L). Pool 3 was targeted just above the upper reference limit of adult men (around 112 μmol/L) [19], pool 4 and 5 were set in the higher concentration range (>177 μmol/L).

### Target values of the five serum pools set by the gold standard method (ID-GC/MS)

The target values (mean ± SD) (ID-GC/MS) of the 5 pools were 35.0 ± 0.004 μmol/L (pool 1), 69.9 ± 0.003 μmol/L (pool 2), 111.8 ± 0.005 μmol/L (pool 3), 296.1 ± 0.013 μmol/L (pool 4) and 933.5 ± 0.011 μmol/L (pool 5).

### Assessment of commutability

To obtain a serum pool, consecutive leftovers of patient samples were added to the already frozen bulk. The influence of freezing and later thawing of the thus obtained pool on commutability was tested on a Cobas 6000 (Roche), as described in the method section. The mean ± SD of the creatinine was 46.3 ± 0.7 μmol/L for the liquid pool and 45.8 ± 0.9 μmol/L for the defrosted pool. The true mean difference between the pools is 0.5 μmol/L with a SD of 0.8 μmol/L and is lying between the predefined equivalence limits of −2.0 and 2.0 μmol/L. The effect of the freeze-thaw cycle is therefore not considered affecting commutability.

### Serum creatinine measurements: CV_i_, bias and error calculations

The Ricos-Fraser criteria were used for assessment of the performance characteristics of the creatinine assays. These criteria are dependent on the within-subject (6.0%) and between-subject (14.7%) variation of creatinine and are commonly used in laboratory practice to subdivide the quality of the performance of an assay into four categories: optimal, desirable, minimal and insufficient (Additional file 1). A good analytical assay should have a low imprecision (CV_i_) compared with the within-subject biological variation and a low bias (B) compared to the target or true value.

#### Intra-laboratory variation CVi

**Figure 1:**
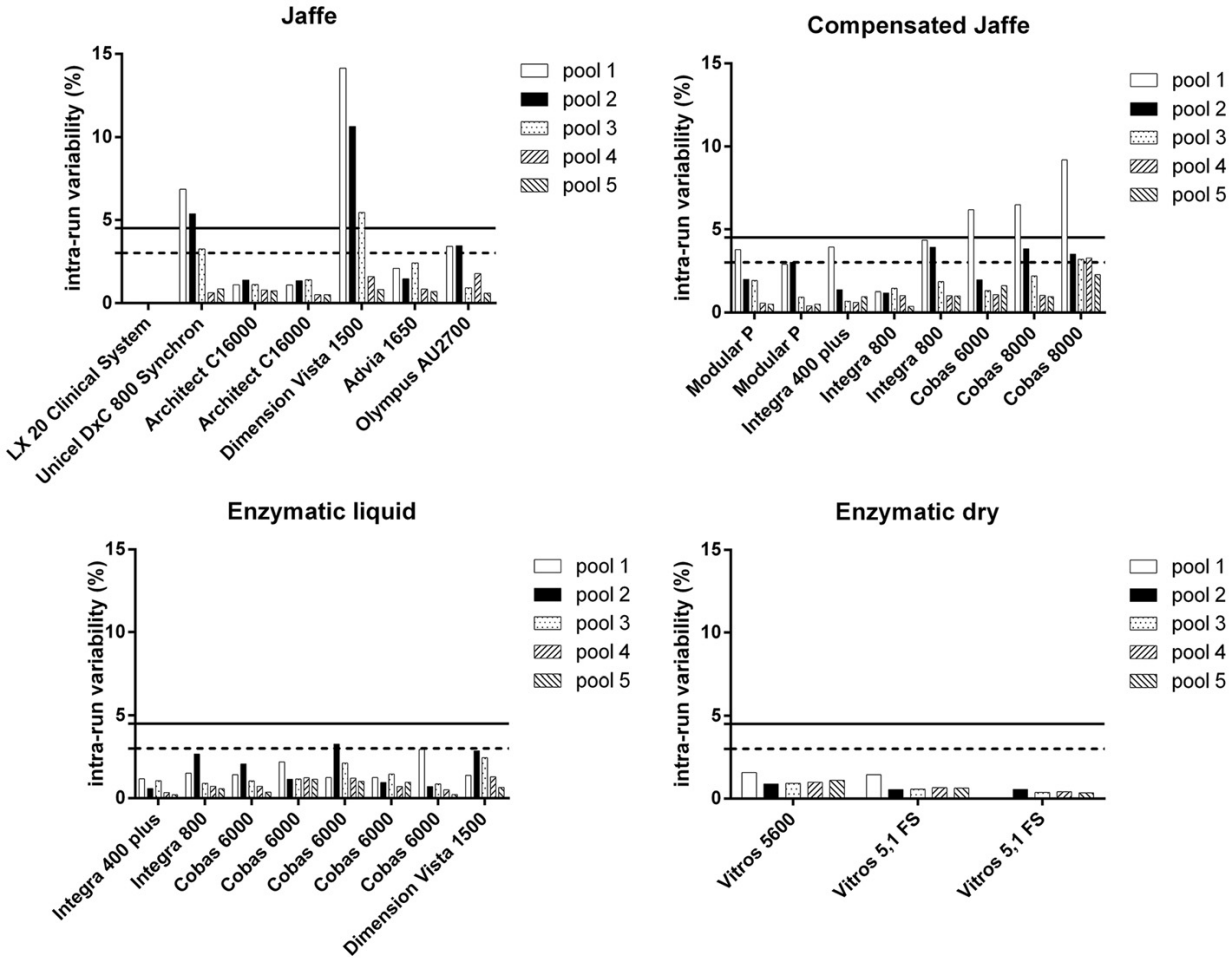
**Intra-run variation of the individual analyzers for each pool according to the type of creatinine assay.** Ricos-Fraser goals are presented. Solid horizontal line: minimal (<4.5%). Dashed horizontal line: desirable (<3.0%). LX20 Clinical System shows no intra-run CV within the reported precision (to the nearest tenth).

In Figure 1 the CV_i_ of the individual analyzers for each pool according to the type of creatinine assay is shown. 81% (21/26) of the analyzer results from pool 1 and 92% (24/26) of the analyzer results from pool 2 met the minimal analytical variation criterion of 4.5% (black line). For pool 3 to 5, >95% of all test results were within the minimal specification criterion. Only Jaffe and/or compensated Jaffe assays did not met the minimal specifications for pool 1 and/or 2 (lower creatinine concentrations). All enzymatic assays were within the minimal specifications.

#### Bias

**Figure 2:**
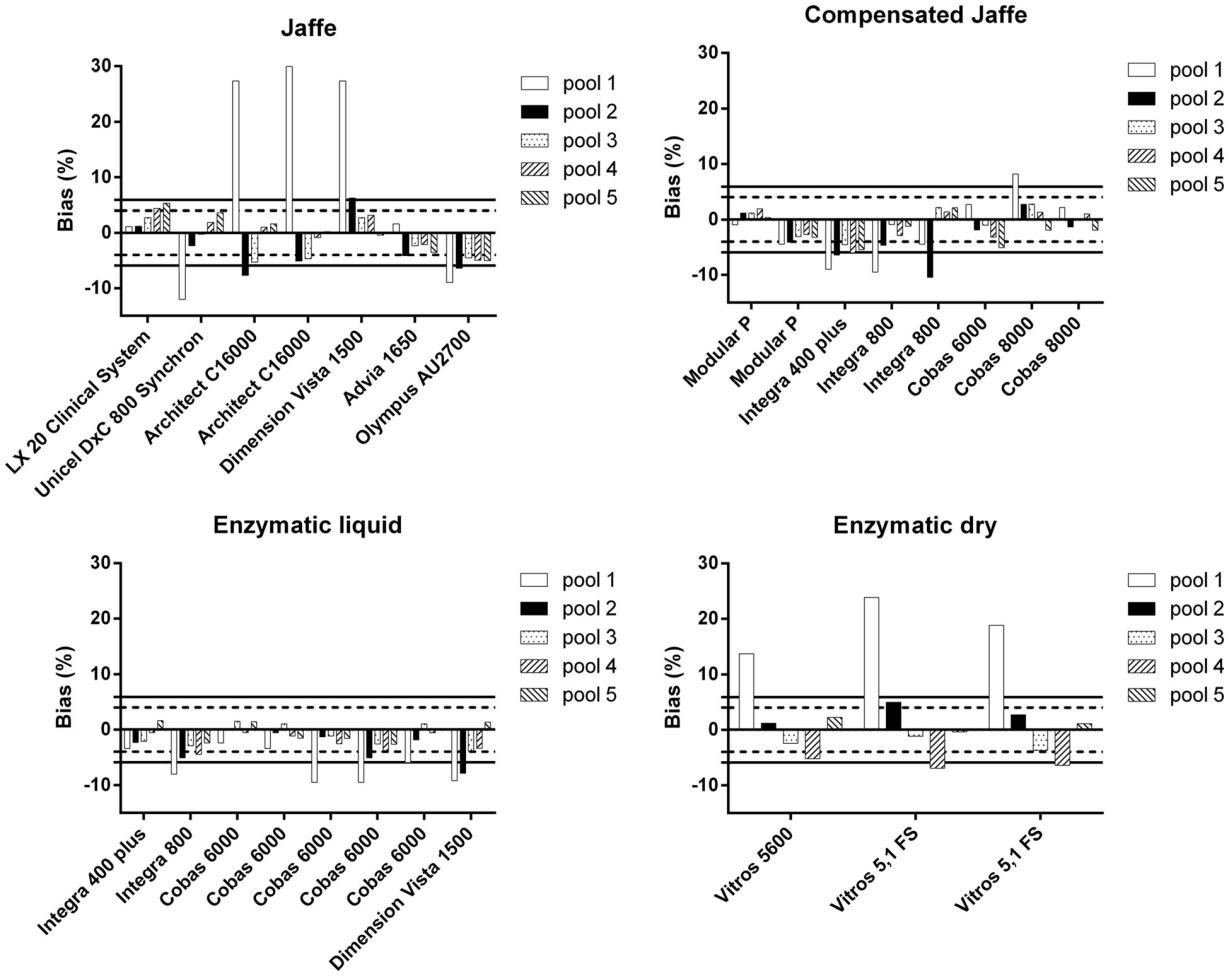
**Bias of the individual analyzers for each pool according to the type of creatinine assay.** Ricos-Fraser goals are presented. Solid horizontal line: minimal (<5.9%). Dashed horizontal line: desirable (<4.0%).

In Figure 2 the bias of the individual analyzers for each pool according to the creatinine assay type is presented. The pool with the lowest creatinine concentration (pool 1) has the largest bias for almost all analyzers. For this pool, 62% (16/26) of the analyzers failed to reach the minimal bias specification of 5.9% (black line). Some Jaffe methods (Dimension Vista 1500 (Siemens) and the two Architect C16000 analyzers (Abbott)) gave very large biases (up to 30%). Also all dry chemistry analyzers (n = 3) (Ortho Clinical Diagnostics Inc.) showed unacceptable positive biases for pool 1 according to Ricos-Fraser. For pool 2, 77% (20/26) of all methods met the minimal bias specifications (<5.9%), but only 50% (13/26) met the desirable bias criterion (<4.0%). For pool 3–5 which are in the adult pathological range, respectively 100%, 88% and 100% of the analyzers met the minimal bias specification. The liquid enzymatic assays (n = 8) have the best score over the whole concentration range, although for pool 1 they all showed negative biases. Moreover, 5 out of 8 of these negative biases are unacceptable.

#### Error calculations (CV_i_ and bias)

**Figure 3:**
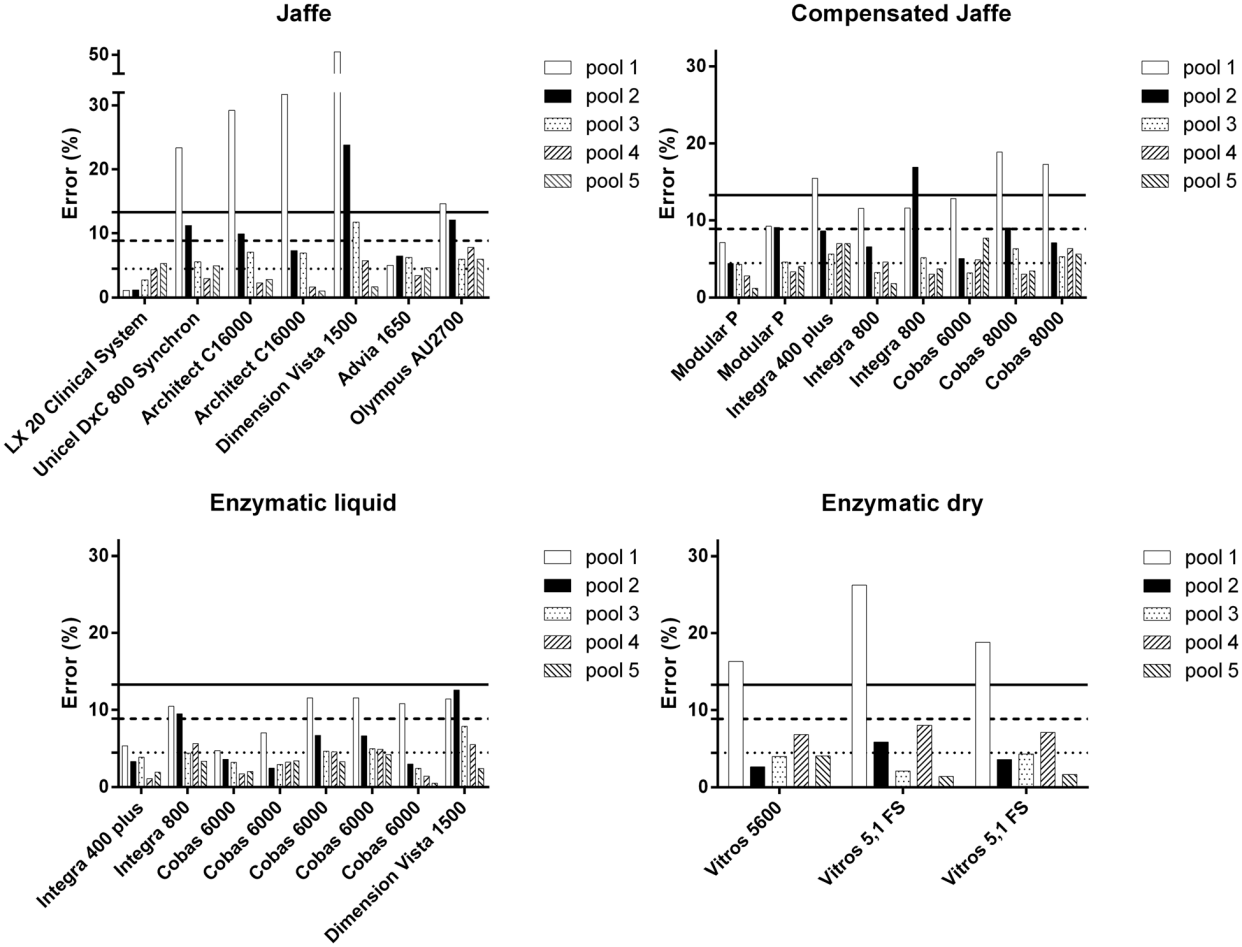
**Error calculations based on CV**_**i**_ **and bias.** Ricos-Fraser total error goals are presented. Solid horizontal line: minimal (<13.3%). Dashed horizontal line: desirable (<8.9%). Dotted horizontal line: optimal (<4.5%).

An error based on CV_i_ and bias (inter-run CV not taken into account) was calculated and compared with the Ricos-Fraser TE (Figure 3). Inter-run experiments were not performed so the actual TEs would presumably be higher than these ‘intermediate’ calculated errors. The calculated errors were compared with the minimal (<13.3%, solid line), desirable (<8.9%, dashed line) and optimal (<4.5%, dotted line) Ricos-Fraser TE categories. Concerning pool 1, the acceptable error (13.3%) was already exceeded for 5 out of 7 Jaffe, for 3 out of 7 compensated Jaffe and for all three dry chemistry analyzers. Extreme errors were calculated for the two Architect C1600 analyzers (Jaffe) (29.2% and 31.7%) and for the Dimension Vista 1500 (Jaffe) (50.7%). For pool 2 the Dimension Vista 1500 (Jaffe) and one Integra 800 (compensated Jaffe) showed insufficient results when compared with the Ricos-Fraser TE criteria. The error calculations for pool 3–5 all fell within the TE budget of Ricos-Fraser.

### eGFR calculations

In Table 2 the minimum and maximum Scr obtained from the individual measurements of each pool in the study can be found. The calculated eGFR values corresponding with these Scr values are also presented. Depending on the Scr assay, a six year old healthy child (length = 116.5 cm) with a Scr value of 35 μmol/L (pool 1) or an 18 year old man with a Scr of 112 μmol/L (pool 3) could be classified in CKD stage G1 or G2. Also a 65 year old woman or man with a Scr concentration of 70 μmol/L (pool 2) could be classified in CKD stage G1 or G2, while a woman of 18 or a man of 65 years with a Scr of 112 μmol/L (pool 3) were classified in stage G2 or G3. A 65 year old women with a Scr value of 112 μmol/L (pool 3) could be classified in stage G3a or G3b. All cases of pool 4 (296 μmol/L), except for the woman aged 65y (G5), corresponds with CKD stage G4. Pool 5 (934 μmol/L) corresponds with CKD stage G5. Overall, in 8/17 cases (47%) a different CKD stage was assigned. In the absence of kidney damage (albuminuria, urine sediment abnormalities, electrolyte and other abnormalities due to tubular disorders, abnormalities detected by histology, structural abnormalities detected by imaging, history of kidney transplantation) Grade 1 and Grade 2 cannot be considered as CKD, but Grade 3 is always considered as CKD, as only the GFR criterion is then relevant for the CKD diagnosis.

## Discussion

In this study we investigated the variability in Scr measurements (CVi, bias and TE) in clinical laboratories in Flanders. The results were grouped according to methodology to reflect how results can vary if patients are tested in different laboratories using various assays. Unacceptable CV_i_, bias and TE were particularly encountered at low creatinine concentrations. At higher creatinine concentrations, CV_i_, bias and TE tended to decrease (Figures 1 and 2). Overall we can say that the enzymatic methods, especially the liquid ones, are performing much better than the Jaffe type assays.

Two participants (LX 20 Clinical System (Jaffe) and Dimension Vista 1500 (Jaffe)) reported results (in mg/dL) which were only accurate to the nearest tenth. Their results should therefore be interpreted with caution. We recommend to report Scr accurate to the nearest hundredth.

The bias in the Jaffe methods is likely due to the non-creatinine chromogens interferences. The compensation made for the mean pseudo-chromogens in the compensated Jaffe assay is an oversimplification because of the variability of these chromogens between different patients and patient groups. Dry chemistry analyzers showed unacceptable positive biases for the lowest concentration pool. The marked differences with the enzymatic liquid assays may suggest that the positive bias in the dry chemistry method could be due to differences in calibration. After Delanghe’s work in 2008 [20] Ortho Clinical Diagnostics Inc. recalibrated its assay resulting in better outcomes in a concentration range >77 μmol/L [5]. According to our results, all dry chemistry assays still gave too large biases for pool 1 (35 μmol/L); for pool 2 (70 μmol/L) the biases are within the minimal bias specification criterion.

The Flanders experience is in line with a Dutch and French multicentric evaluation performed in 2012 and 2013: the (compensated) Jaffe creatinine methods did not reach the desirable specifications of NKDEP at normal levels of creatinine [3,21]. The two groups also showed that enzymatic methods on the other hand have an excellent traceability to the ID-GC/MS reference method for creatinine [5] except for the dry chemistry methods. In this project, the calculated errors of the liquid enzymatic assays were within the minimal TE criterion of 13.3% for all five studied creatinine concentrations. We demonstrated that attention should be paid when dry chemistry methods are used to determine low Scr values. Pool 1 (35 μmol/L) largely exceeded the minimal TE. This has major consequences for the assessment of renal function and CKD staging (which are mostly estimated by creatinine-based equations) in the pediatric population. Also for other patients with low Scr values (like anorectic or cancer patients or the elderly) and even for the healthy population, one should be aware of the shortcomings of the creatinine assay used.

One could argue that the Ricos-Fraser approach is not applicable at very low substance concentrations. The literature provides little information on the performance characteristics of creatinine when using this parameter as a diagnostic test in neonates and children or in adult patients with low serum creatinine. However, based on the pediatric within-subject variation of 6.4% and the pediatric between-subject variation of 20.1% published by Andersen et al. [22], the minimal specifications are 4.8% for CV_i_, 7.9% for bias and 15.8% for TE (Ricos-Fraser approach). These minimal specifications are very similar to those published by Ricos et al. [18] (Additional file 1). Information about performance specifications based on clinical needs is also scarce. A paper by Schwartz et al. [23] considers an increase of Scr from 26.5 to 35.4 μmol/L, or a change of 33% of major clinical significance in children. This implies that the TE should be ≤20-30% in the pediatric creatinine range. According to KDIGO (Kidney Disease–Improving Global Outcomes) a change in GFR by 5 mL/min/1.73 m^2^ is considered as clinically relevant [24]. For a person with a GFR of 100 mL/min/1.73 m^2^ this corresponds with a TE of 5%. Based on the biological variability of the GFR and based on our experience, a change in GFR of at least 10% is clinically relevant. When using the updated Schwartz equation [8] for calculating the eGFR in the pediatric population, an analytical bias of 5% or a TE of 10% on the Scr concentration leads to the same bias or TE on the calculated eGFR value. Based on the above mentioned arguments, we also used the criteria of Ricos-Fraser for pool 1 (35 μmol/L) with a target value in the pediatric concentration range.

Our study has several strengths. We studied 26 routine instrumental methods, representing 13 different types of analyzers from 6 different manufacturers which delivered test results of five pools covering a wide creatinine concentration range from 35 to 934 μmol/L. Moreover, fresh frozen serum samples were used instead of non-commutable lyophilized control materials. However, only three labs working with Vitros instruments and five different labs working with enzymatic liquid assays were involved in the study.

Our data illustrated that the minimum and maximum Scr values determined for the same pool in different labs with various assays could lead to a large spread of calculated eGFR values (Table 2) especially in the lower Scr concentration range (pool 1–3). The eGFR values calculated with the reported Scr values might even classify patients in other CKD stages.

## Conclusions

Overall, we can conclude that although most assays claim to be traceable to ID-GC/MS, large inter-assay differences still exist. The inaccuracy in the lower concentration range is of particular concern and may lead to clinical misinterpretation especially in children and in patients with muscle wasting and consequently low creatinine values like in anorectic or cancer patients, or in the elderly. Enzymatic assays, especially the liquid ones, lead to less variability in Scr measurements than Jaffe type methods. We therefore advocate a more general use of the enzymatic creatinine assays, but nevertheless attention should still be paid in the lower Scr concentration range. Further efforts should be made to improve the calibration of commercialized creatinine assays so that the CV_i_, bias and TE are kept within the minimal criteria of Ricos-Fraser.

## Additional file

Additional file 1:
**Imprecision, bias and total error criteria according to Ricos-Fraser.** CV_w_: within subject biological variation = 6.0% and CV_b_: between subject variation = 14.7% [18].

## Abbreviations

eGFR: Estimated glomerular filtration rate
Scr: Serum creatinine
CKD: Chronic kidney disease
NKDEP: National kidney disease education program
ID-GC/MS: Isotope dilution gas chromatography/mass spectrometry
MDRD: Modification of diet in renal disease
CKD-EPI: Chronic kidney disease epidemiology collaboration
JCTMC: Joint committee on traceability in laboratory medicine
E: Enzymatic
AP-K: Alkaline picrate kinetic
AP-RB: Alkaline picrate rate blanked
C: Compensated
NC: Non-compensated
G: Grade
SD: Standard deviation
CV_i_: Intra-run coefficient of variation
B: Bias
TE: Total error
KDIGO: Kidney disease – improving global outcomes
