# Supplementary material for: Routine serum creatinine measurements: how well do we perform?
Source: BMC Nephrol. 2015 Feb 14;16:21. doi: 10.1186/s12882-015-0012-x (PMC4358903; doi:10.1186/s12882-015-0012-x)
Supplement: Additional file 1: — Imprecision, bias and total error criteria according to Ricos-Fraser. CVw: within subject biological variation = 6.0% and CVb: between subject variation = 14.7% [18]. [file 12882_2015_12_MOESM1_ESM.doc]

**ADDITIONAL FILES**

**Additional file 1**. Imprecision, bias and total error criteria according to Ricos-Fraser. CV_w_: within subject biological variation = 6.0% and CV_b_: between subject variation = 14.7% .

| **Criterion** | **Imprecision (CV)(%)** | | **Bias (B)(%)** | | **Total Error (TE)(%)** | |
| --- | --- | --- | --- | --- | --- | --- |
| Insufficient | ≥0.75CV_w_ | ≥4.5 | ≥0.375(CV_w_² + CV_b_²)^0.5^ | ≥5.9 | 1.65CV+B | ≥13.3 |
| Minimal | <0.75 CV_w_ | <4.5 | <0.375(CV_w_² + CV_b_²)^0.5^ | <5.9 | 1.65CV+B | <13.3 |
| Desirable | <0.5 CV_w_ | <3.0 | <0.25(CV_w_² + CV_b_²)^0.5^ | <4.0 | 1.65CV+B | <8.9 |
| Optimal | <0.25 CV_w_ | <1.5 | <0.125(CV_w_² + CV_b_²)^0.5^ | <2.0 | 1.65CV+B | <4.5 |
